# Dietary and Physical Activity Behaviors Among High School Students — Youth Risk Behavior Survey, United States, 2025

**DOI:** 10.15585/mmwr.su7501a2

**Published:** 2026-10-15

**Authors:** Caitlin Merlo, Ryan Saelee, Shannon L. Michael, Ann Goding Sauer, Sandra J. Kiplagat, Ellen Wenders, Carmen Ashley, Catherine N. Rasberry

**Affiliations:** ^1^Division of Adolescent and School Health, National Center for Chronic Disease Prevention and Health Promotion, CDC, Atlanta, Georgia; ^2^Division of Nutrition, Physical Activity, and Obesity, National Center for Chronic Disease Prevention and Health Promotion, CDC, Atlanta, Georgia; ^3^U.S. Public Health Service, Rockville, Maryland

## Abstract

Adolescence is a critical period for establishing healthy dietary and physical activity behaviors, which support growth, development, and chronic disease prevention. However, most adolescents do not meet national guidelines for healthy eating and daily physical activity, and previous data indicated declines in these behaviors. This report uses nationally representative data from the 2025 Youth Risk Behavior Survey to update estimates of dietary and physical activity behaviors among U.S. high school students overall and by sex, race and ethnicity, and grade. In addition, 2-year comparisons (2023 versus 2025) of these behaviors were examined. In 2025, during the 7 days before the survey was administered, 39% of high school students ate fruit ≥1 time per day, 63% ate vegetables ≥1 time per day, 27% ate breakfast daily, 30% had not drunk sugar-sweetened soda or pop, 51% had not drunk sports drinks, and 58% drank ≥3 glasses per day of plain water. In addition, approximately 25% of students met aerobic guidelines (were physically active for ≥60 minutes per day on all 7 days), 51% met muscle-strengthening guidelines (exercised to strengthen muscles on ≥3 days per week), 16% met both aerobic and muscle-strengthening guidelines, 20% attended physical education classes on all 5 school days, and 58% played on at least one sports team during the 12 months before the survey. From 2023 to 2025, overall increases were observed for eating fruit ≥1 time per day, eating vegetables ≥1 time per day, and drinking ≥3 glasses per day of plain water. Sports team participation also increased; however, students attending physical education class on all 5 school days decreased. Despite certain improvements since 2023, the 2025 prevalence of most healthy dietary behaviors and physical activity behaviors remained low. Understanding the current data can guide actions to create environments that support healthy behaviors in schools, communities, and at home and might help adolescents meet national recommendations for healthy eating and physical activity.

## Introduction

Adolescence is a critical period for establishing healthy dietary and physical activity behaviors that support growth and development, reduce the risk for chronic conditions such as heart disease and Type 2 diabetes (*1**,**2*), and are linked to better mental health and academic achievement (*3**,**4*). Despite these benefits, most adolescents do not meet recommended guidelines for healthy eating, such as eating enough vegetables and fruits and avoiding sugar-sweetened beverages, or for physical activity, including engaging in daily aerobic activity and muscle-strengthening activities at least 3 days a week (*2**,**5**,**6*).

Previously released data from the national Youth Risk Behavior Survey (YRBS) found that in the decade from 2013 to 2023, most adolescent dietary and physical activity behaviors either worsened or did not improve, with trends varying across demographic groups (*5*). Healthy People 2030, a set of national objectives for improving the health and well-being of all persons in the United States, continues to include and prioritize objectives related to dietary and physical activity health behaviors as well as evidence-based practices (e.g., physical education and participation in school breakfast programs) that support healthy development across multiple settings (*7*). Promoting these behaviors and practices is consistent with more than 2 decades of school-based research on approaches to support healthy eating and physical activity (*8**–**10*).

Continuing to monitor adolescent dietary and physical activity behaviors and differences across demographic groups is foundational to improving these behaviors and associated health outcomes among adolescents. Using recent nationally representative YRBS data, this report provides 2025 estimates of dietary and physical activity behaviors among adolescents overall and by sex, race and ethnicity, and grade level; identifies changes in these behaviors from 2023 to 2025; and presents supplemental analyses of selected dietary behaviors to provide additional context related to the newly released 2025–2030 Dietary Guidelines for Americans (DGA) (*6*). These findings can help prioritize actions that support healthy eating and physical activity in the places where adolescents spend their time, including in schools, in community spaces, and at home.

## Methods

### Data Source

This report includes data from the 2023 (N = 20,103) and 2025 (N = 10,710) YRBSs, the two most recent of the cross-sectional, school-based surveys conducted biennially since 1991. Each survey year, CDC collects data from a nationally representative sample of students from public and private schools, including parochial schools, in grades 9–12 in the 50 U.S. states and the District of Columbia. Additional information about YRBS sampling, data collection, response rates, and processing is available in the overview report of this supplement (*11*). The prevalence estimates for all YRBS variables among U.S. high school students overall and stratified by demographic characteristics are available at https://youthonline.cdc.gov. The full YRBS questionnaire, datasets, and documentation are available at https://www.cdc.gov/yrbs/index.html. This study was approved by institutional review boards at CDC and ICF, the survey contractor, and was conducted consistent with applicable federal law and CDC policy.*

### Measures

Questions, response options, and analytic coding for the six dietary and five physical activity behaviors included in this report are provided (Table 1). Dietary behaviors included 1) eating fruit ≥1 time per day, 2) eating vegetables ≥1 time per day, 3) eating breakfast daily, 4) not drinking soda or pop (excluding diet soda or diet pop), 5) not drinking sports drinks (excluding low-calorie sports drinks), and 6) drinking ≥3 glasses per day of plain water, all during the 7 days before the survey was administered. In addition, two dietary measures were recoded for supplemental analyses to provide additional context related to DGA, which recommend eating two servings of fruit and three servings of vegetables each day for a 2000-calorie diet, adjusting as necessary for personal caloric needs (*6*). Although YRBS assesses frequency of intake rather than servings eaten, data were analyzed to estimate the prevalence of eating fruit ≥2 times per day and vegetables ≥3 times per day during the past 7 days to more closely align with DGA. The percentage of missing responses for dietary behaviors ranged from 4.3% (eating fruit ≥1 time per day) to 36.9% (not drinking sports drinks). To reduce respondent effort, students in schools selected for both the national and site-level YRBS samples were administered only one questionnaire: either the national or site-specific questionnaire. Because site-specific questionnaires might not include all questions on the national YRBS questionnaire, some students included in the national sample were not asked certain dietary and physical activity questions. These procedures contributed to the higher levels of missing data observed for certain measures.

**TABLE 1:** Questions, response options, and analytic coding for included dietary and physical activity behavior variables — Youth Risk Behavior Survey, United States, 2023 and 2025

Variable	Question	Response option	Analytic coding
**Dietary behavior**			
Ate fruit ≥1 time per day	During the past 7 days, how many times did you eat fruit? (Do not count fruit juice.)	I did not eat fruit during the past 7 days, 1–3 times during the past 7 days, 4–6 times during the past 7 days,1 time per day, 2 times per day, 3 times per day, or ≥4 times per day	≥1 time per day versus <1 time per day
Ate vegetables ≥1 time per day	During the past 7 days, how many times did you eat • green salad? • potatoes? (Do not count French fries, fried potatoes, or potato chips.) • carrots? • other vegetables? (Do not count green salad, potatoes, or carrots.)	I did not eat (green salad)/(potatoes)/(carrots)/(other vegetables) during the past 7 days, 1–3 times during the past 7 days, 4–6 times during the past 7 days, 1 time per day, 2 times per day, 3 times per day, or ≥4 times per day	≥1 time per day versus <1 time per day
Ate breakfast daily	During the past 7 days, on how many days did you eat breakfast?	0 days, 1 day, 2 days, 3 days, 4 days, 5 days, 6 days, or 7 days	7 days versus ≤6 days
Did not drink soda or pop	During the past 7 days, how many times did you drink a can, bottle, or glass of soda or pop, such as Coke, Pepsi, or Sprite? (Do not count diet soda or diet pop.)	I did not drink soda or pop during the past 7 days, 1–3 times during the past 7 days, 4–6 times during the past 7 days, 1 time per day, 2 times per day, 3 times per day, or ≥4 times per day	0 times during the past 7 days versus ≥1 time during the past 7 days
Did not drink a sports drink	During the past 7 days, how many times did you drink a can, bottle, or glass of a sports drink, such as Gatorade or Powerade? (Do not count low-calorie sports drinks such as Propel or G2.)	I did not drink sports drinks during the past 7 days, 1–3 times during the past 7 days, 4–6 times during the past 7 days, 1 time per day, 2 times per day, 3 times per day, or ≥4 times per day	0 times during the past 7 days versus ≥1 time during the past 7 days
Drank ≥3 glasses per day of plain water	During the past 7 days, how many times did you drink a bottle or glass of plain water? (Count tap, bottled, and unflavored sparkling water.)	I did not drink water during the past 7 days, 1–3 times during the past 7 days, 4–6 times during the past 7 days, 1 time per day, 2 times per day, 3 times per day, or ≥4 times per day	≥3 times per day versus <3 times per day
**Physical activity behavior**			
Were physically active for a total of ≥60 minutes per day on all 7 days (met the guideline for aerobic activity)	During the past 7 days, on how many days were you physically active for a total of at least 60 minutes per day? (Add up all the time you spent in any kind of physical activity that increased your heart rate and made you breathe hard some of the time.)	0 days, 1 day, 2 days, 3 days, 4 days, 5 days, 6 days, or 7 days	7 days versus <7 days
Did exercises to strengthen or tone muscles on ≥3 days (met the guideline for muscle-strengthening activity)	During the past 7 days, on how many days did you do exercises to strengthen or tone your muscles, such as push-ups, sit-ups, or weightlifting?	0 days, 1 day, 2 days, 3 days, 4 days, 5 days, 6 days, or 7 days	≥3 days versus <3 days
Met both aerobic and muscle-strengthening guidelines	(See “were physically active for a total of ≥60 minutes per day on all 7 days” and “did exercises to strengthen or tone muscles on ≥3 days.”)	NA	Physically active for ≥60 minutes per day on all 7 days and did exercises to strengthen or tone muscles on ≥3 days versus physically active for <60 minutes per day on all 7 days or did exercises to strengthen or tone muscles on <3 days
Attended physical education classes on all 5 school days	In an average week when you are in school, on how many days do you go to physical education (PE) classes?	0 days, 1 day, 2 days, 3 days, 4 days, or 5 days	5 days versus <5 days
Played on at least 1 sports team	During the past 12 months, on how many sports teams did you play? (Count any teams run by your school or community groups.)	0 teams, 1 team, 2 teams, or ≥3 teams	≥1 team versus <1 team

**Abbreviation:** NA = not applicable.

Physical activity behaviors included 1) being physically active for a total of ≥60 minutes per day on all 7 days during the 7 days before the survey (met the guideline for aerobic activity), 2) exercising to strengthen or tone muscles on ≥3 days during the 7 days before the survey (met the guideline for muscle-strengthening activity), 3) meeting both aerobic and muscle-strengthening guidelines in the past 7 days, 4) attending physical education classes on all 5 days during an average school week (daily physical education), and 5) playing on at least one sports team during the 12 months before the survey. The percentage of missing responses for physical activity behaviors ranged from 4.8% (being physically active for a total of ≥60 minutes per day on all 7 days) to 37.3% (exercising to strengthen or tone muscles on ≥3 days).

Demographic variables included sex (female or male), race and ethnicity (American Indian or Alaska Native [AI/AN], Asian, Black or African American [Black], Hispanic or Latino [Hispanic], Middle Eastern or North African [ME/NA], Native Hawaiian or Pacific Islander, White, or multiracial [selected more than one racial category]), and grade (9, 10, 11, or 12). Persons of Hispanic origin might be of any race but are categorized as Hispanic; all racial groups are non-Hispanic.

### Analysis

The prevalence and 95% CIs were estimated for each dietary and physical activity behavior overall and by demographic characteristics for 2023 and 2025. Pairwise design-adjusted *t* tests were used to compare differences in prevalence by demographic characteristics and year (2023 versus 2025); differences were considered statistically significant at p<0.05. Corrections for multiple comparisons were not applied because the analyses were descriptive and hypothesis generating, and each statistical test addressed a distinct research question and was interpreted independently. All estimates were calculated using Taylor series linearization. Prevalence estimates with denominators <30 were considered statistically unreliable and therefore were suppressed. All analyses accounted for complex sampling design and weighting and were conducted using SAS-callable SUDAAN (version 11.0.4; RTI International). 

## Results

### Dietary Behaviors

In 2025, 38.9% of high school students had eaten fruit ≥1 time per day, 62.5% had eaten vegetables ≥1 time per day, 27.0% had eaten breakfast daily, 30.4% had not drunk soda or pop, 50.8% had not drunk sports drinks, and 58.3% had drunk ≥3 glasses per day of plain water (Table 2). A higher prevalence of male students than female students had eaten breakfast every day (31.3% versus 22.5%) and had drunk ≥3 glasses per day of plain water (60.7% versus 55.5%). In contrast, a higher prevalence of female students than male students had not drunk sports drinks (59.1% versus 43.1%). In general, a higher prevalence of Asian students than students in other racial and ethnic groups engaged in healthy eating behaviors, with certain exceptions. For example, ME/NA students had the highest prevalence of having eaten fruit ≥1 time per day (58.1%). Although differences by grade were also observed for three of the six dietary behaviors (having eaten fruit ≥1 time per day, eaten breakfast daily, and not drunk a soda or pop), a clear pattern was not evident.

**TABLE 2:** Percentage of high school students with selected dietary behaviors, by demographic characteristics — Youth Risk Behavior Survey, United States, 2025*

Characteristic	Ate fruit ≥1 time per day^†^	Ate vegetables ≥1 time per day^§^	Ate breakfast daily^¶^	Did not drink sugar-sweetened soda or pop**	Did not drink sports drink^††^	Drank ≥3 glasses per day of plain water^§§^
	% (95% CI)	% (95% CI)	% (95% CI)	% (95% CI)	% (95% CI)	% (95% CI)
**Overall**	**38.9 (36.6–41.3)**	**62.5 (60.1–64.9)**	**27.0 (25.0–29.1)**	**30.4 (27.9–33.0)**	**50.8 (47.1–54.5)**	**58.3 (54.5–61.9)**
**Sex**						
Female	38.6 (35.4–41.8)	61.7 (58.4–64.9)	22.5 (20.1–25.0)	31.3 (28.4–34.4)	59.1 (54.8–63.3)	55.5 (51.4–59.5)
Male	39.3 (36.9–41.8)	63.5 (60.8–66.1)	31.3 (28.4–34.4)	29.3 (26.3–32.6)	43.1 (39.9–46.3)	60.7 (56.7–64.6)
**Race and ethnicity^¶¶^**						
AI/AN	43.8 (25.4–64.1)	61.6 (42.4–77.8)	36.6 (22.6–53.3)	31.6 (16.8–51.3)	42.8 (25.2–62.4)	47.5 (25.1–71.1)
Asian	41.6 (36.9–46.4)	77.6 (72.1–82.2)	36.8 (29.5–44.8)	44.4 (40.4–48.4)	68.6 (60.4–75.8)	68.0 (59.9–75.1)
Black or African American	30.8 (27.1–34.7)	51.4 (47.2–55.6)	21.7 (19.3–24.1)	28.4 (25.4–31.6)	45.3 (39.8–51.0)	53.4 (47.2–59.4)
Hispanic or Latino	37.7 (35.1–40.5)	58.8 (56.3–61.2)	24.9 (22.2–27.7)	27.8 (25.0–30.7)	47.5 (43.3–51.6)	60.6 (57.1–64.0)
ME/NA	58.1 (41.6–73.1)	75.3 (58.1–87.0)	31.8 (23.0–42.0)	33.9 (24.0–45.5)	56.0 (39.9–70.8)	68.5 (52.4–81.1)
NH/PI	42.6 (22.6–65.2)	—***	11.7 (2.2–43.3)	—***	—***	—***
White	40.4 (36.7–44.2)	64.9 (61.3–68.3)	28.3 (25.4–31.3)	30.1 (26.3–34.2)	51.3 (46.4–56.1)	56.9 (52.8–60.9)
Multiracial	40.2 (33.1–47.8)	65.6 (58.0–72.5)	25.1 (20.9–29.8)	32.7 (25.4–40.9)	52.8 (45.5–60.1)	55.8 (46.9–64.3)
**Grade**						
9	41.8 (38.7–44.9)	62.9 (59.4–66.3)	29.3 (26.8–31.9)	28.1 (25.4–30.9)	48.2 (43.3–53.1)	57.8 (53.8–61.8)
10	40.0 (36.3–43.9)	61.0 (57.7–64.3)	27.9 (25.0–31.1)	31.2 (27.8–34.8)	49.3 (45.2–53.4)	57.5 (54.1–60.9)
11	38.6 (35.7–41.5)	63.3 (59.6–66.9)	25.3 (22.2–28.8)	31.1 (27.7–34.8)	51.9 (46.7–57.0)	59.0 (54.0–63.7)
12	34.5 (30.7–38.6)	63.0 (59.5–66.3)	24.7 (21.5–28.3)	31.4 (27.8–35.1)	54.7 (48.4–60.8)	59.0 (52.0–65.6)

**Abbreviations:** AI/AN = American Indian or Alaska Native; ME/NA = Middle Eastern or North African; NH/PI = Native Hawaiian or Pacific Islander; YRBS = Youth Risk Behavior Survey.

* N = 10,710 respondents. The total number (N) of students answering each question varied. Data might be missing because 1) students in schools selected for both national and site-specific YRBS samples completed only one questionnaire; therefore, when the site-specific questionnaire was administered, it might not have included all questions on the national YRBS; 2) the student did not answer the question; or 3) the response was set to missing because of an out-of-range response or logical inconsistency. Prevalence estimates in each category are calculated based on nonmissing data.

^†^ N = 10,252 respondents. As determined by *t* test analysis with Taylor series linearization (p<0.05), no differences were detected by sex; Asian students significantly differed from Black or African American (Black) and ME/NA students; Black students significantly differed from Hispanic or Latino (Hispanic), ME/NA, White, and multiracial students; Hispanic students significantly differed from ME/NA students; ME/NA students significantly differed from White and multiracial students; and grade 9, 10, and 11 students significantly differed from grade 12 students.

^§^ N = 8,781 respondents. As determined by *t* test analysis with Taylor series linearization (p<0.05), no differences were detected by sex; Asian students significantly differed from Black, Hispanic, White, and multiracial students; Black students significantly differed from Hispanic, ME/NA, White, and multiracial students; Hispanic students significantly differed from ME/NA and White students; and no differences by grade were detected.

^¶^ N = 10,172 respondents. As determined by *t* test analysis with Taylor series linearization (p<0.05), female students significantly differed from male students; AI/AN students significantly differed from NH/PI students; Asian students significantly differed from Black, Hispanic, NH/PI, White, and multiracial students; Black students significantly differed from Hispanic, ME/NA, and White students; Hispanic students significantly differed from White students; and grade 9 students significantly differed from grade 11 and 12 students.

** N = 8,758 respondents. As determined by *t* test analysis with Taylor series linearization (p<0.05), no differences were detected by sex; Asian students significantly differed from Black, Hispanic, White, and multiracial students; and grade 9 students significantly differed from grade 12 students.

^††^ N = 6,763 respondents. As determined by *t* test analysis with Taylor series linearization (p<0.05), female students significantly differed from male students; AI/AN students significantly differed from Asian students; Asian students significantly differed from Black, Hispanic, White, and multiracial students; and no differences by grade were detected.

^§§^ N = 7,847 respondents. As determined by *t* test analysis with Taylor series linearization (p<0.05), female students significantly differed from male students; Asian students significantly differed from Black, White, and multiracial students; Black students significantly differed from Hispanic students; and no differences by grade were detected.

^¶¶^ Persons of Hispanic origin might be of any race but are categorized as Hispanic; all racial groups are non-Hispanic.

*** Prevalence estimates with a denominator of <30 were considered statistically unreliable and therefore were suppressed.

From 2023 to 2025, significant changes were observed overall for three of the six dietary behaviors examined (Table 3). The prevalence of students who had eaten fruit ≥1 time per day increased overall and among female, male, and White students, as well as among students in grades 9, 10, and 11. The prevalence of students who had eaten vegetables ≥1 time per day increased overall and among female, male, and Hispanic students, and among students in grades 9, 11, and 12. The prevalence of students who had drunk ≥3 glasses per day of plain water increased overall and among male, Asian, and Hispanic students, and among students in grades 9 and 11. Although no changes in the full sample were observed for eating breakfast daily, this behavior did significantly increase from 2023 to 2025 for AI/AN students.

**TABLE 3:** Changes from 2023 to 2025 in the percentage of high school students with selected dietary behaviors, by demographic characteristics and survey year — Youth Risk Behavior Survey, United States*

Behavior	2023	2025	Change from 2023 to 2025^†^
	% (95% CI)	% (95% CI)	
**Ate fruit ≥1 time per day**	N = 19,027	N = 10,252	**Increase**
**Overall**	**32.9 (31.1–34.8)**	**38.9 (36.6–41.3)**	
**Sex**			
Female	31.4 (29.6–33.3)	38.6 (35.4–41.8)	Increase
Male	34.4 (32.0–36.9)	39.3 (36.9–41.8)	Increase
**Race and ethnicity^§^**			
AI/AN	22.6 (14.3–33.7)	43.8 (25.4–64.1)	No change
Asian	36.9 (30.1–44.2)	41.6 (36.9–46.4)	No change
Black or African American	28.8 (24.8–33.1)	30.8 (27.1–34.7)	No change
Hispanic or Latino	34.0 (30.7–37.4)	37.7 (35.1–40.5)	No change
ME/NA	—^¶^	58.1 (41.6–73.1)	—^¶^
NH/PI	36.3 (27.2–46.6)	42.6 (22.6–65.2)	No change
White	33.4 (31.1–35.7)	40.4 (36.7–44.2)	Increase
Multiracial	32.3 (27.1–38.0)	40.2 (33.1–47.8)	No change
**Grade**			
9	34.0 (31.3–36.7)	41.8 (38.7–44.9)	Increase
10	33.8 (31.3–36.4)	40.0 (36.3–43.9)	Increase
11	33.0 (30.1–36.1)	38.6 (35.7–41.5)	Increase
12	30.6 (27.5–33.8)	34.5 (30.7–38.6)	No change
**Ate vegetables ≥1 time per day**	N = 14,938	N = 8,781	**Increase**
**Overall**	**57.5 (55.5–59.6)**	**62.5 (60.1–64.9)**	
**Sex**			
Female	56.4 (53.7–58.9)	61.7 (58.4–64.9)	Increase
Male	58.6 (56.4–60.9)	63.5 (60.8–66.1)	Increase
**Race and ethnicity^§^**			
AI/AN	66.2 (49.5–79.7)	61.6 (42.4–77.8)	No change
Asian	71.4 (65.9–76.3)	77.6 (72.1–82.2)	No change
Black or African American	48.8 (44.3–53.2)	51.4 (47.2–55.6)	No change
Hispanic or Latino	54.3 (51.4–57.1)	58.8 (56.3–61.2)	Increase
ME/NA	—^¶^	75.3 (58.1–87.0)	—^¶^
NH/PI	52.8 (39.8–65.4)	—**	—**
White	61.0 (58.6–63.4)	64.9 (61.3–68.3)	No change
Multiracial	56.8 (50.4–63.0)	65.6 (58.0–72.5)	No change
**Grade**			
9	57.1 (53.7–60.4)	62.9 (59.4–66.3)	Increase
10	57.7 (55.2–60.2)	61.0 (57.7–64.3)	No change
11	58.2 (55.2–61.2)	63.3 (59.6–66.9)	Increase
12	57.1 (53.1–61.1)	63.0 (59.5–66.3)	Increase
**Ate breakfast daily**	N = 14,268	N = 10,172	**No change**
**Overall**	**27.4 (25.4–29.6)**	**27.0 (25.0–29.1)**	
**Sex**			
Female	22.4 (20.4–24.6)	22.5 (20.1–25.0)	No change
Male	32.2 (29.6–34.8)	31.3 (28.4–34.4)	No change
**Race and ethnicity^§^**			
AI/AN	13.6 (6.9–25.1)	36.6 (22.6–53.3)	Increase
Asian	32.1 (26.0–39.0)	36.8 (29.5–44.8)	No change
Black or African American	23.8 (20.3–27.6)	21.7 (19.3–24.1)	No change
Hispanic or Latino	24.3 (20.9–28.0)	24.9 (22.2–27.7)	No change
ME/NA	—^¶^	31.8 (23.0–42.0)	—^¶^
NH/PI	22.4 (11.3–39.8)	11.7 (2.2–43.3)	No change
White	30.5 (27.8–33.3)	28.3 (25.4–31.3)	No change
Multiracial	27.0 (21.0–34.1)	25.1 (20.9–29.8)	No change
**Grade**			
9	29.0 (26.3–31.8)	29.3 (26.8–31.9)	No change
10	27.6 (24.7–30.7)	27.9 (25.0–31.1)	No change
11	25.1 (22.5–27.8)	25.3 (22.2–28.8)	No change
12	27.9 (24.8–31.2)	24.7 (21.5–28.3)	No change
**Did not drink sugar-sweetened soda or pop**	N = 15,183	N = 8,758	**No change**
**Overall**	**30.9 (28.7–33.3)**	**30.4 (27.9–33.0)**	
**Sex**			
Female	33.2 (30.9–35.6)	31.3 (28.4–34.4)	No change
Male	28.6 (26.1–31.2)	29.3 (26.3–32.6)	No change
**Race and ethnicity^§^**			
AI/AN	22.1 (12.6–35.9)	31.6 (16.8–51.3)	No change
Asian	39.8 (34.6–45.3)	44.4 (40.4–48.4)	No change
Black or African American	32.1 (28.1–36.4)	28.4 (25.4–31.6)	No change
Hispanic or Latino	30.0 (26.7–33.6)	27.8 (25.0–30.7)	No change
ME/NA	—^¶^	33.9 (24.0–45.5)	—^¶^
NH/PI	34.4 (24.4–46.0)	—**	—**
White	30.0 (27.8–32.3)	30.1 (26.3–34.2)	No change
Multiracial	30.8 (25.9–36.1)	32.7 (25.4–40.9)	No change
**Grade**			
9	28.7 (25.5–32.2)	28.1 (25.4–30.9)	No change
10	31.5 (29.4–33.8)	31.2 (27.8–34.8)	No change
11	30.2 (26.5–34.3)	31.1 (27.7–34.8)	No change
12	33.1 (30.2–36.2)	31.4 (27.8–35.1)	No change
**Did not drink a sports drink**	N = 11,909	N = 6,763	**No change**
**Overall**	**48.5 (45.4–51.6)**	**50.8 (47.1–54.5)**	
**Sex**			
Female	58.3 (54.9–61.5)	59.1 (54.8–63.3)	No change
Male	39.3 (36.2–42.6)	43.1 (39.9–46.3)	No change
**Race and ethnicity^§^**			
AI/AN	46.1 (33.0–59.7)	42.8 (25.2–62.4)	No change
Asian	64.6 (58.1–70.5)	68.6 (60.4–75.8)	No change
Black or African American	43.0 (34.1–52.4)	45.3 (39.8–51.0)	No change
Hispanic or Latino	46.7 (42.9–50.6)	47.5 (43.3–51.6)	No change
ME/NA	—^¶^	56.0 (39.9–70.8)	—^¶^
NH/PI	41.3 (24.1–60.8)	—**	—**
White	49.4 (45.7–53.1)	51.3 (46.4–56.1)	No change
Multiracial	47.1 (40.0–54.3)	52.8 (45.5–60.1)	No change
**Grade**			
9	42.2 (38.3–46.2)	48.2 (43.3–53.1)	No change
10	47.8 (44.7–50.9)	49.3 (45.2–53.4)	No change
11	48.4 (43.9–52.9)	51.9 (46.7–57.0)	No change
12	55.7 (51.6–59.8)	54.7 (48.4–60.8)	No change
**Drank ≥3 glasses per day of plain water**	N = 13,584	N = 7,847	**Increase**
**Overall**	**53.6 (51.6–55.7)**	**58.3 (54.5–61.9)**	
**Sex**			
Female	51.0 (48.8–53.3)	55.5 (51.4–59.5)	No change
Male	56.1 (53.7–58.5)	60.7 (56.7–64.6)	Increase
**Race and ethnicity^§^**			
AI/AN	51.7 (38.5–64.8)	47.5 (25.1–71.1)	No change
Asian	57.4 (50.4–64.2)	68.0 (59.9–75.1)	Increase
Black or African American	49.8 (44.4–55.3)	53.4 (47.2–59.4)	No change
Hispanic or Latino	52.4 (49.7–55.1)	60.6 (57.1–64.0)	Increase
ME/NA	—^¶^	68.5 (52.4–81.1)	—^¶^
NH/PI	73.4 (52.4–87.4)	—**	—**
White	54.4 (52.2–56.6)	56.9 (52.8–60.9)	No change
Multiracial	57.0 (50.8–63.1)	55.8 (46.9–64.3)	No change
**Grade**			
9	52.4 (49.3–55.4)	57.8 (53.8–61.8)	Increase
10	55.5 (52.7–58.2)	57.5 (54.1–60.9)	No change
11	51.0 (47.5–54.5)	59.0 (54.0–63.7)	Increase
12	55.9 (53.1–58.7)	59.0 (52.0–65.6)	No change

**Abbreviations:** AI/AN = American Indian or Alaska Native; ME/NA = Middle Eastern or North African; NH/PI = Native Hawaiian or Pacific Islander; YRBS = Youth Risk Behavior Survey.

* N = 20,103 respondents for 2023 and N = 10,710 respondents for 2025. The total number (N) of students answering each question varied. Data might be missing because 1) students in schools selected for both national and site-specific YRBS samples completed only one questionnaire; therefore, when the site-specific questionnaire was administered, it might not have included all questions on the national YRBS; 2) the student did not answer the question; or 3) the response was set to missing because of an out-of-range response or logical inconsistency. Prevalence estimates in each category are calculated based on nonmissing data.

^†^ As determined by *t* test analysis, p<0.05.

^§^ Persons of Hispanic or Latino (Hispanic) origin might be of any race but are categorized as Hispanic; all racial groups are non-Hispanic.

^¶^ Data for ME/NA students were not collected in 2023.

** Prevalence estimates with a denominator of <30 were considered statistically unreliable and therefore were suppressed.

### Supplemental Analysis of Fruit and Vegetable Consumption

In 2025, 28.1% of students had eaten fruit ≥2 times per day and 16.2% had eaten vegetables ≥3 times per day (Supplementary Table). Differences by race and ethnicity were similar to the primary analysis of having eaten fruit ≥1 time per day and having eaten vegetables ≥1 time per day. The prevalence of students in grade 9 who had eaten fruit ≥2 times per day (31.9%) and vegetables ≥3 times per day (18.4%) was higher than the prevalence for students in grades 11 and 12.

From 2023 to 2025, significant increases were observed in the prevalence of students who had eaten fruit ≥2 times per day overall and among female, male, Hispanic, White, and multiracial students, as well as students in each grade (Supplementary Table). The prevalence of students who had eaten vegetables ≥3 times per day increased overall and among female, male, Hispanic, and White students and among students in grades 9 and 12.

### Physical Activity Behaviors

In 2025, 25.0% of students had been physically active for ≥60 minutes per day on all 7 days, 50.6% had exercised to strengthen or tone muscles on ≥3 days per week, 16.1% had met both aerobic and muscle-strengthening guidelines, 19.8% had attended physical education classes on all 5 days in an average school week, and 57.6% had played on at least one sports team (Table 4). A higher prevalence of male students than female students had been physically active for ≥60 minutes per day on all 7 days (31.5% versus 18.2%), had exercised to strengthen or tone muscles on ≥3 days per week (61.2% versus 39.4%), had met both aerobic and muscle-strengthening physical activity guidelines (22.0% versus 10.0%), had attended physical education classes on all 5 days (23.4% versus 16.3%), and had played on at least one sports team (60.5% versus 54.6%). Differences by race and ethnicity did not indicate a consistent pattern across physical activity behaviors. Significant grade-level differences were observed for each physical activity behavior; when differences were identified, students in lower grade levels reported higher engagement in physical activity behaviors than students in higher grade levels.

**TABLE 4:** Percentage of high school students with selected physical activity behaviors, by demographic characteristics — Youth Risk Behavior Survey, United States, 2025*

Characteristic	Were physically active for a total of ≥60 minutes per day on all 7 days^†^	Did exercises to strengthen or tone muscles on ≥3 days^§^	Met both aerobic and muscle-strengthening guidelines^¶^	Went to physical education classes on all 5 days**	Played on at least one sports team^††^
	% (95% CI)	% (95% CI)	% (95% CI)	% (95% CI)	% (95% CI)
**Overall**	**25.0 (23.4–26.7)**	**50.6 (47.7–53.4)**	**16.1 (13.1–19.5)**	**19.8 (15.6–24.9)**	**57.6 (53.0–62.1)**
**Sex**					
Female	18.2 (16.8–19.8)	39.4 (36.6–42.2)	10.0 (8.0–12.5)	16.3 (12.3–21.2)	54.6 (49.4–59.8)
Male	31.5 (29.6–33.5)	61.2 (57.7–64.5)	22.0 (18.3–26.2)	23.4 (18.7–28.7)	60.5 (55.7–65.2)
**Race and ethnicity^§§,¶¶^**					
AI/AN	22.9 (11.9–39.5)	38.8 (21.6–59.3)	7.5 (2.8–18.4)	13.9 (5.6–30.3)	40.6 (22.3–62.0)
Asian	19.0 (14.2–24.8)	48.8 (40.6–57.0)	11.8 (7.4–18.3)	12.0 (8.0–17.6)	47.3 (39.9–54.9)
Black or African American	22.5 (19.4–25.9)	49.2 (45.0–53.5)	12.1 (9.0–15.9)	27.6 (20.3–36.3)	55.4 (50.4–60.3)
Hispanic or Latino	21.2 (19.4–23.2)	48.3 (44.0–52.6)	13.3 (10.6–16.5)	23.9 (18.4–30.4)	51.5 (47.6–55.4)
ME/NA	22.3 (11.9–38.1)	65.0 (53.8–74.7)	15.3 (7.1–29.9)	28.9 (16.7–45.1)	61.7 (49.8–72.4)
White	28.2 (26.0–30.5)	51.7 (47.5–55.9)	19.1 (15.4–23.4)	16.7 (12.7–21.5)	62.1 (55.8–68.0)
Multiracial	26.8 (21.2–33.3)	52.3 (45.7–58.8)	17.0 (12.4–23.0)	17.6 (12.7–24.0)	62.5 (54.8–69.6)
**Grade**					
9	26.1 (23.1–29.3)	53.4 (48.9–57.9)	17.3 (13.9–21.3)	29.2 (23.0–36.3)	61.2 (55.9–66.3)
10	27.5 (24.5–30.8)	52.5 (48.5–56.5)	17.9 (14.3–22.1)	18.5 (14.2–23.7)	60.2 (54.4–65.7)
11	22.7 (20.4–25.0)	48.8 (45.3–52.3)	14.2 (11.1–18.0)	15.9 (11.7–21.2)	57.3 (51.1–63.3)
12	23.3 (20.6–26.2)	46.9 (43.1–50.8)	14.6 (11.3–18.6)	14.7 (10.7–19.8)	50.7 (45.5–55.9)

**Abbreviations:** AI/AN = American Indian or Alaska Native; ME/NA = Middle Eastern or North African; YRBS = Youth Risk Behavior Survey.

* N = 10,710 respondents. The total number (N) of students answering each question varied. Data might be missing because 1) students in schools selected for both national and site-specific YRBS samples completed only one questionnaire; therefore, when the site-specific questionnaire was administered, it might not have included all questions on the national YRBS; 2) the student did not answer the question; or 3) the response was set to missing because of an out-of-range response or logical inconsistency. Prevalence estimates in each category are calculated based on nonmissing data.

^†^ N = 10,197 respondents. As determined by *t* test analysis with Taylor series linearization (p<0.05), female students significantly differed from male students; Asian students significantly differed from White and multiracial students; Black or African American (Black) students significantly differed from White students; Hispanic or Latino (Hispanic) students significantly differed from White and multiracial students; and grade 9 and 10 students significantly differed from grade 11 students.

^§^ N = 6,717 respondents. As determined by *t* test analysis with Taylor series linearization (p<0.05), female students significantly differed from male students; ME/NA students significantly differed from AI/AN, Asian, Black, Hispanic, and White students; grade 9 students significantly differed from grade 11 and 12 students; and grade 10 students significantly differed from grade 12 students.

^¶^ N = 9,433 respondents. As determined by *t* test analysis with Taylor series linearization (p<0.05), female students significantly differed from male students; AI/AN students significantly differed from White and multiracial students; Asian students significantly differed from White students; Black students significantly differed from White and multiracial students; Hispanic students significantly differed from White students; grade 9 students significantly differed from grade 11 students; and grade 10 students significantly differed from grade 11 and 12 students.

** N = 8,892 respondents. As determined by *t* test analysis with Taylor series linearization (p<0.05), female students significantly differed from male students; AI/AN students significantly differed from Black students; Asian students significantly differed from Black, Hispanic, ME/NA, White, and multiracial students; Black students significantly differed from White and multiracial students; Hispanic students significantly differed from White students; grade 9 students significantly differed from grade 10, 11, and 12 students; and grade 10 students significantly differed from grade 12 students.

^††^ N = 7,109 respondents. As determined by *t* test analysis with Taylor series linearization (p<0.05), female students significantly differed from male students; AI/AN students significantly differed from White and multiracial students; Asian students significantly differed from ME/NA, White, and multiracial students; Hispanic students significantly differed from ME/NA, White, and multiracial students; and grade 9, 10, and 11 students significantly differed from grade 12 students.

^§§^ Persons of Hispanic origin might be of any race but are categorized as Hispanic; all racial groups are non-Hispanic.

^¶¶^ Prevalence estimates with a denominator <30 were considered statistically unreliable and were suppressed; therefore, estimates for Native Hawaiian or Pacific Islander students are not presented.

From 2023 to 2025, significant changes were observed in two physical activity behaviors (Table 5). The prevalence of students attending physical education classes daily decreased overall and among female, AI/AN, White, and multiracial students, and those in grade 10. In contrast, the prevalence of playing on at least one sports team increased overall and among female students.

**TABLE 5:** Changes from 2023 to 2025 in the percentage of high school students with physical activity behaviors, by demographic characteristics and survey year — Youth Risk Behavior Survey, United States*

Behavior	2023	2025	Change from 2023 to 2025^†^
	% (95% CI)	% (95% CI)	
**Were physically active for a total of ≥60 minutes per day on all 7 days**	N = 18,876	N = 10,197	**No change**
**Overall**	**24.6 (22.8–26.5)**	**25.0 (23.4–26.7)**	
**Sex**			
Female	16.6 (15.0–18.3)	18.2 (16.8–19.8)	No change
Male	32.2 (30.0–34.4)	31.5 (29.6–33.5)	No change
**Race and ethnicity^§^**			
AI/AN	31.9 (14.5–56.4)	22.9 (11.9–39.5)	No change
Asian	15.6 (11.6–20.7)	19.0 (14.2–24.8)	No change
Black or African American	21.5 (19.3–24.0)	22.5 (19.4–25.9)	No change
Hispanic or Latino	20.1 (17.3–23.3)	21.2 (19.4–23.2)	No change
ME/NA	—^¶^	22.3 (11.9–38.1)	—^¶^
NH/PI	29.3 (19.1–42.1)	—**	—**
White	29.0 (26.7–31.4)	28.2 (26.0–30.5)	No change
Multiracial	26.3 (21.6–31.5)	26.8 (21.2–33.3)	No change
**Grade**			
9	26.9 (24.4–29.6)	26.1 (23.1–29.3)	No change
10	26.4 (23.5–29.6)	27.5 (24.5–30.8)	No change
11	23.7 (21.1–26.4)	22.7 (20.4–25.0)	No change
12	21.0 (18.3–24.1)	23.3 (20.6–26.2)	No change
**Did exercises to strengthen or tone muscles on ≥3 days**	N = 11,197	N = 6,717	**No change**
**Overall**	**51.1 (48.0–54.1)**	**50.6 (47.7–53.4)**	
**Sex**			
Female	37.2 (34.3–40.2)	39.4 (36.6–42.2)	No change
Male	63.8 (60.7–66.8)	61.2 (57.7–64.5)	No change
**Race and ethnicity^§^**			
AI/AN	44.2 (31.2–58.1)	38.8 (21.6–59.3)	No change
Asian	45.6 (39.5–51.7)	48.8 (40.6–57.0)	No change
Black or African American	46.7 (42.5–51.0)	49.2 (45.0–53.5)	No change
Hispanic or Latino	48.8 (45.2–52.4)	48.3 (44.0–52.6)	No change
ME/NA	—^¶^	65.0 (53.8–74.7)	—^¶^
NH/PI	52.6 (39.1–65.7)	—**	—**
White	54.3 (49.5–59.0)	51.7 (47.5–55.9)	No change
Multiracial	56.2 (49.8–62.5)	52.3 (45.7–58.8)	No change
**Grade**			
9	55.3 (51.5–59.1)	53.4 (48.9–57.9)	No change
10	52.1 (47.5–56.6)	52.5 (48.5–56.5)	No change
11	48.8 (44.0–53.6)	48.8 (45.3–52.3)	No change
12	47.9 (44.0–51.7)	46.9 (43.1–50.8)	No change
**Met both aerobic and muscle-strengthening guidelines**	N = 17,042	N = 9,433^††^	**No change**
**Overall**	**16.5 (14.0–19.3)**	**16.1 (13.1–19.5)**	
**Sex**			
Female	9.4 (7.7–11.3)	10.0 (8.0–12.5)	No change
Male	23.5 (20.2–27.1)	22.0 (18.3–26.2)	No change
**Race and ethnicity^§^**			
AI/AN	14.3 (9.1–21.9)	7.5 (2.8–18.4)	No change
Asian	9.1 (6.3–12.9)	11.8 (7.4–18.3)	No change
Black or African American	12.2 (9.2–16.0)	12.1 (9.0–15.9)	No change
Hispanic or Latino	14.4 (11.1–18.4)	13.3 (10.6–16.5)	No change
ME/NA	—^¶^	15.3 (7.1–29.9)	—^¶^
NH/PI	27.3 (17.9–39.1)	—**	—**
White	19.5 (16.1–23.3)	19.1 (15.4–23.4)	No change
Multiracial	20.4 (16.1–25.5)	17.0 (12.4–23.0)	No change
**Grade**			
9	17.5 (14.4–20.9)	17.3 (13.9–21.3)	No change
10	18.2 (14.6–22.4)	17.9 (14.3–22.1)	No change
11	16.2 (13.0–19.9)	14.2 (11.1–18.0)	No change
12	14.0 (11.3–17.2)	14.6 (11.3–18.6)	No change
**Went to physical education classes on all 5 days**	N = 13,876	N = 8,892	**Decrease**
**Overall**	**27.4 (21.9–33.6)**	**19.8 (15.6–24.9)**	
**Sex**			
Female	23.1 (18.3–28.9)	16.3 (12.3–21.2)	Decrease
Male	31.4 (24.9–38.6)	23.4 (18.7–28.7)	No change
**Race and ethnicity^§^**			
AI/AN	34.9 (23.0–49.0)	13.9 (5.6–30.3)	Decrease
Asian	20.6 (12.8–31.3)	12.0 (8.0–17.6)	No change
Black or African American	24.4 (17.2–33.3)	27.6 (20.3–36.3)	No change
Hispanic or Latino	30.9 (23.4–39.7)	23.9 (18.4–30.4)	No change
ME/NA	—^¶^	28.9 (16.7–45.1)	—^¶^
NH/PI	35.2 (19.8–54.5)	—**	—**
White	26.2 (20.5–33.0)	16.7 (12.7–21.5)	Decrease
Multiracial	28.9 (20.9–38.6)	17.6 (12.7–24.0)	Decrease
**Grade**			
9	39.5 (30.5–49.3)	29.2 (23.0–36.3)	No change
10	30.3 (23.5–38.1)	18.5 (14.2–23.7)	Decrease
11	21.4 (16.2–27.7)	15.9 (11.7–21.2)	No change
12	17.1 (12.9–22.3)	14.7 (10.7–19.8)	No change
**Played on at least one sports team**	N = 12,964	N = 7,109	**Increase**
**Overall**	**51.9 (48.9–54.9)**	**57.6 (53.0–62.1)**	
**Sex**			
Female	48.1 (45.0–51.2)	54.6 (49.4–59.8)	Increase
Male	55.7 (52.0–59.4)	60.5 (55.7–65.2)	No change
**Race and ethnicity^§^**			
AI/AN	57.0 (44.7–68.4)	40.6 (22.3–62.0)	No change
Asian	46.0 (37.5–54.8)	47.3 (39.9–54.9)	No change
Black or African American	49.8 (45.7–53.9)	55.4 (50.4–60.3)	No change
Hispanic or Latino	47.3 (43.6–51.0)	51.5 (47.6–55.4)	No change
ME/NA	—^¶^	61.7 (49.8–72.4)	—^¶^
NH/PI	56.6 (44.6–67.9)	—**	—**
White	56.2 (52.0–60.3)	62.1 (55.8–68.0)	No change
Multiracial	57.0 (51.2–62.5)	62.5 (54.8–69.6)	No change
**Grade**			
9	57.2 (53.1–61.2)	61.2 (55.9–66.3)	No change
10	54.2 (50.2–58.2)	60.2 (54.4–65.7)	No change
11	50.2 (45.9–54.5)	57.3 (51.1–63.3)	No change
12	46.1 (41.1–51.3)	50.7 (45.5–55.9)	No change

**Abbreviations:** AI/AN = American Indian or Alaska Native; ME/NA = Middle Eastern or North African; NH/PI = Native Hawaiian or Pacific Islander; YRBS = Youth Risk Behavior Survey.

* N = 20,103 respondents for 2023 and N = 10,710 respondents for 2025. The total number (N) of students answering each question varied. Data might be missing because 1) students in schools selected for both national and site-specific YRBS samples completed only one questionnaire; therefore, when the site-specific questionnaire was administered, it might not have included all questions on the national YRBS; 2) the student did not answer the question; or 3) the response was set to missing because of an out-of-range response or logical inconsistency. Prevalence estimates in each category are calculated based on nonmissing data.

^†^ As determined by *t* test analysis, p<0.05.

^§^ Persons of Hispanic or Latino (Hispanic) origin might be of any race but are categorized as Hispanic; all racial groups are non-Hispanic.

^¶^ Data for ME/NA students were not collected in 2023.

** Prevalence estimates with a denominator of <30 were considered statistically unreliable and therefore were suppressed.

^††^ Participants were classified as meeting the combined guideline only if they met both components and as not meeting the combined guideline if they did not meet either component, regardless of missing data for the other component. Participants were classified as missing if both components were missing or if one component was missing and the observed component was met.

## Discussion

Healthy eating and regular physical activity are important contributors to adolescents’ physical and mental well-being. From 2023 to 2025, improvements were observed overall in four of the 11 dietary and physical activity behaviors examined, while only one behavior, daily physical education, decreased. Ongoing national surveillance through YRBSs can help identify population-level trends and possible differences across demographic groups that can guide priority setting, implementation, and evaluation of strategies to improve adolescent nutrition and physical activity.

Although the prevalence of students who had eaten fruit daily increased to approximately 40% and those who had eaten vegetables daily increased to approximately 60%, eating these foods only once daily is a low threshold. To benefit more from healthy eating, adolescents’ fruit and vegetable consumption should be more than once daily (*6*). The supplemental analysis assessing higher intake frequencies demonstrates that, overall, in 2025, only 28% of students had eaten fruit ≥2 times per day and 16% had eaten vegetables ≥3 times per day, with the prevalence of both increasing since 2023. Although these findings represent frequency of intake rather than amount eaten, they suggest that many high school students might not eat recommended amounts (*12*).

The DGA recommend water and other unsweetened beverages for hydration. These findings indicate an increase in the prevalence of students who had drunk ≥3 glasses per day of plain water in 2025 compared with 2023. Consistent with a previous report (*5*), the prevalence of students who did not drink soda or sports drinks was unchanged. The 2025 data indicate that approximately seven in 10 students had drunk soda or pop, and approximately half of students had drunk sports drinks, during the past 7 days. Reducing sugary beverage consumption could help reduce adolescents’ consumption of added sugars (*1*).

The prevalence of three physical activity behaviors remained consistent from 2023 to 2025, including meeting the guideline for aerobic activity, meeting the guideline for muscle-strengthening activity, and meeting both guidelines. Data indicate that in 2025, one in four high school students met the guideline for aerobic activity, approximately half met the guideline for muscle strengthening, and 16% of students met both. These findings suggest that many students are not meeting national physical activity guidelines, which could have implications for physical and mental well-being and long-term health outcomes (*2*,*13*).

The prevalence of two physical activity behaviors changed from 2023 to 2025. First, the prevalence of students attending physical education class on all 5 school days decreased overall and among demographic subgroups, with approximately one in five high school students (19.8%) meeting this recommended standard in 2025. Previous research indicates that students who attended physical education classes on all 5 days were twice as likely to meet both aerobic and muscle-strengthening guidelines as those who did not (*14*), suggesting the potential value of physical education for providing opportunities for physical activity during the school day and building the knowledge, skills, and confidence to stay active in other settings and throughout life (*9*,*10*). Second, the prevalence for sports team participation increased from 52% in 2023 to 58% in 2025. This finding might be important because sports team participation can provide adolescents with structured opportunities for physical activity, skill development, confidence-building, and social connection beyond physical education class (*15*).

In 2025, differences by demographic subgroups were observed across all 11 dietary and physical activity behaviors analyzed. In addition, from 2023 to 2025, within certain demographic subgroups, several healthy eating behaviors increased, including fruit, vegetable, breakfast, and water consumption. No decreases were noted in any of the behaviors among demographic subgroups except daily physical education, which decreased among several demographic groups. In contrast, sports team participation increased among female students, which might reflect growing efforts by schools and communities to support female involvement in sports (*16*).

Findings from this study highlight potential opportunities for continued efforts across federal, state, and local levels to help adolescents meet recommendations for healthy eating and physical activity. National objectives and guidelines, including Healthy People 2030 and DGA, provide broader goals for improving these behaviors, whereas federal and state programs can support implementation of evidence-based strategies in the settings where adolescents spend their time, including home, communities, and schools (*17*). For example, CDC’s Healthy Schools Program supports 19 state-level entities and one tribal nation (https://www.cdc.gov/healthy-schools/partners/index.html) that in turn work with school districts and schools to implement school health activities in the context of the Whole School, Whole Community, Whole Child (WSCC) framework. This framework is used to coordinate among schools, communities, and families to address the needs of the whole child (https://www.cdc.gov/whole-school-community-child/about/index.html). Within WSCC, CDC supports schools to implement a range of activities designed to improve school nutrition environments and offer increased opportunities for physical activity both within and beyond the school day. One of these recipients, the Missouri Department of Elementary and Secondary Education, has used CDC support to implement policies that support Comprehensive School Physical Activity Programs (CSPAP) across the state, especially in rural areas. The CSPAP framework helps schools increase physical activity opportunities before, during, and after school (*9*). In addition to including physical education and sports teams, CSPAP also supports implementation of school-level physical activity policies, active commuting to and from school, and physical activity lessons in the classroom. Engagement of families and communities, as well as staff member involvement in CSPAP design, can help tailor activities to the students who need them and the capacity and priorities of the school.

Schools can also implement various strategies to increase student access to and consumption of nutritious foods at school. For example, providing school nutrition staff members with professional development to prepare healthy and appealing meals, engaging students in school gardens, and providing taste tests of new healthy menu items increases consumption of healthy foods (*8*). In addition, offering a grab-and-go breakfast when students arrive, or during morning breaks, can increase breakfast access for students by eliminating the need to arrive before the school day begins (*18*). YRBS data can complement these implementation efforts by helping state and local partners identify behaviors and populations that might warrant additional attention and by providing population-level context for program planning and evaluation.

## Limitations

General limitations for the YRBS are available in the overview report of this supplement (*11*). The findings in this report are subject to at least four additional limitations. First, questions about fruit and vegetable intake measure frequency of consumption rather than number of servings. Therefore, determining whether students are meeting the current dietary recommendations is not possible. Second, the physical education (PE) question assessed attendance only during an average school week and did not account for PE policies at the individual schools, semester-based enrollment, or completion of PE requirements, which might have affected reported PE attendance. However, the PE measure has demonstrated strong test-retest reliability (*19*). Third, socioeconomic measures known to be associated with dietary and physical activity behaviors and opportunities were not collected by YRBS and therefore could not be examined. Finally, analytic denominators varied across dietary and physical activity variables because of item-specific missing data. Missing data resulted from student nonresponse, invalid responses, and survey design procedures used to minimize respondent fatigue when schools were selected for both national and site-level YRBS samples; in such instances, certain students were not asked all national YRBS questions. If responders and nonresponders systematically differed, this could have affected the generalizability of estimates.

## Future Directions

Although decades of research have examined policies, practices, and programs to improve healthy eating and physical activity behaviors among children and adolescents, research that examines which strategies work best among high school students could help practitioners better plan interventions (*4*,*8*,*9*). Future research and program evaluation could focus on understanding contextual factors associated with implementation of strategies overall and among populations of high school students with a lower prevalence of healthy behaviors. Additional research could explore the extent to which data on the number of times particular foods were eaten aligns with number of servings, enhancing researchers’ ability to understand the extent to which adolescents’ dietary behaviors align per day with national recommendations.

## Conclusion

Although the frequency of fruit and vegetable intake, the prevalence of students who drank ≥3 glasses per day of plain water, and sports team participation improved from 2023 to 2025, the prevalence of most healthy dietary and physical activity behaviors examined in this study remains low. Interventions that increase access to and opportunities for healthy food and beverage choices and physical activity could help support healthier behaviors. Data on observed differences in behaviors across demographic subgroups could help public health professionals better tailor interventions and identify groups that might benefit from additional support. Overall, findings can help decision makers prioritize actions to create healthy environments, including in schools, so that adolescents can meet national recommendations for healthy eating and physical activity.

## Footnotes

* 45 C.F.R. part 46.114; 21 C.F.R. part 56.114.
